# Comparison of Mutations Induced by Different Doses of Fast-Neutron Irradiation in the M_1_ Generation of Sorghum (*Sorghum bicolor*)

**DOI:** 10.3390/genes15080976

**Published:** 2024-07-24

**Authors:** Na Yuan, Shuaiqiang Liang, Ling Zhou, Xingxing Yuan, Chunhong Li, Xin Chen, Han Zhao

**Affiliations:** 1 Institute of Industrial Crops, Jiangsu Key Laboratory for Horticultural Crop Genetic Improvement, Jiangsu Academy of Agricultural Sciences, Nanjing 210014, China; thefuries@163.com (N.Y.); yxx@jaas.ac.cn (X.Y.); 19960015@jaas.ac.cn (C.L.); 2 Institute of Crop Germplasm and Biotechnology, Provincial Key Laboratory of Agrobiology, Jiangsu Academy of Agricultural Sciences, Nanjing 210014, China; liangshuaiqiang@126.com (S.L.); zlingxiaoyao@163.com (L.Z.)

**Keywords:** sorghum, fast-neutron irradiation, mutation, whole-genome re-sequencing, genetic variation

## Abstract

Sorghum is an important C_4_ crop with various food and nonfood uses. Although improvements through hybridization and selection have been exploited, the introduction of genetic variation and the development of new genotypes in sorghum are still limited. Fast-neutron (FN) mutagenesis is a very effective method for gene functional studies and to create genetic variability. However, the full spectrum of FN-induced mutations in sorghum is poorly understood. To address this, we generated an FN-induced mutant population from the inbred line ‘BTx623’ and sequenced 40 M_1_ seedlings to evaluate the mutagenic effects of FNs on sorghum. The results show that each line had an average of 43.7 single-base substitutions (SBSs), 3.7 InDels and 35.15 structural variations (SVs). SBSs accounted for approximately 90.0% of the total number of small mutations. Among the eight treatment groups, FN irradiation at a dose of 19 Gy generated the highest number of mutations. The ratio of transition/transversion ranged from 1.77 to 2.21, and the G/C to A/T transition was the most common substitution in all mutant lines. The distributions of the identified SBSs and InDels were similar and uneven across the genome. An average of 3.63 genes were mutated in each mutant line, indicating that FN irradiation resulted in a suitable density of mutated genes, which can be advantageous for improving elite material for one specific or a few traits. These results provide a basis for the selection of the suitable dose of mutagen and new genetic resources for sorghum breeding.

## 1. Introduction

Sorghum (*Sorghum bicolor* L. Moench) is one of the oldest cereal crops in the world. It is not only an important grain crop, but also a raw material with great potential for the traditional brewing industry, animal husbandry and the new renewable-energy industry [1,2]. In recent years, the market demand for new varieties of short-stalked, early maturing grain and silage sorghum has been increasing, and sorghum breeding aimed purely at high yields can no longer meet the diverse needs of the market [3]. Therefore, cultivating new materials to meet the diverse needs of the market has become a new goal for sorghum breeders.

Since the first release of an improved tobacco cultivar in the 1930s, mutation breeding has become an effective strategy for crop improvement [4,5]. Mutants not only can be directly bred into cultivars or provide parental materials for breeding, but also are indispensable and important materials for functional genomics research in the post-genomic era [6]. Commonly used mutagenesis approaches include physical mutagenesis, chemical mutagenesis, T-DNA insertion, transposon insertion, etc. Ethyl methanesulfonate (EMS) is the most commonly used chemical mutagen for creating a mutagenized population [7]. It has the advantages of low cost, simplicity of operation and high frequency of point mutations in the DNA, but the derived mutations are genetically less stable and impose a great risk to researcher and environmental health. Ionizing radiation mutagenesis is another popular method for developing mutant varieties [8]. Among the 3402 mutant cultivars officially registered in the FAO/IAEA mutant variety database (http://mvgs.iaea.org) (accessed on 10 December 2023), 2116 were obtained by ionizing radiation. In particular, γ-rays are the most commonly used ionizing radiation source in mutation breeding programs. However, with the development of high-energy cyclotrons and research reactors, heavy ions and neutrons, which have a significantly higher LET than γ-rays, are increasingly being used for mutation breeding [9,10]. Fast neutrons (FNs) are very efficient mutagens in plants, reacting with protons (H^+^) and other heavy nuclei to produce γ-rays and charged particles, which in turn bombard the DNA in the cell nucleus. FN mutagenesis usually creates deletions from few bases to several million bases (Mb) and chromosome rearrangements in the genome, and it is easy to generate FN-treated lines and deletion libraries [11]. It has been successfully used in many plants, including *Arabidopsis thaliana*, rice, soybean, pea, *Medicago truncatula* and peanut [12,13,14,15,16,17,18,19].

The radiation dose is an important factor affecting the mutagenic efficiency [20,21]. By analyzing the biological effect induced by different neutron dosages, Xu et al. [22] observed that a low dosage of FN irradiation (0.38 Gy) significantly promoted the germination and radicle growth of the M_1_ generation of peas, and higher dosages (1.66, 4.33 Gy) reduced the plant height. Based on the germination rates and the relative variation frequencies, Zhang et al. [23] showed 40~45 Gy is the appropriate dose for the FN mutagenesis of the azuki bean. Normally, a dose resulting in 50% survival of the M_1_ plants (lethal dose 50 or LD50) is expected to have the highest mutation frequency and is considered an appropriate dose [24]. In the last decade, with the rapid development of next-generation sequencing technologies, the genome mutation profile has become another important index for evaluating the mutagenic effects of FN irradiation. For example, a genome-wide analysis of mutations induced by FN irradiation in *A. thaliana* found that the frequency of FN-induced single-base substitution mutations was higher than that of deletion mutations and that there were more single-base deletions than large base deletions [13]. Similar to *A. thaliana*, SBSs constituted the most abundant mutation type in an FN-mutagenized population of rice [14]. Researchers also found that FN-induced deletions mutate the largest number of genes. However, these studies were usually carried out in the M_3_ and subsequent generations; so, less is known about the original number and distribution of genomic mutations in the M_1_ plants induced by different FN doses.

In sorghum, EMS-induced mutant libraries have been established in diverse genetic backgrounds such as ‘BTx623’, ‘Tian C-1’, ‘Jinliang No.5’ and ‘V4B’ [25,26,27,28,29]. EMS mostly generates one type of mutation, whereas other mutagenesis approaches such as FN irradiation can induce a diversity of mutations, including single-base substitutions (SBSs), deletions, insertions, inversions, translocations and duplications [13,14,15]. A broad spectrum of mutant alleles, including loss-of-function, partial loss-of-function, and gain-of-function alleles, is highly desirable for functional genomic studies. However, other mutagenesis approaches such as FN irradiation have not been reported in sorghum, which limits the discovery of novel genes and functional elements controlling diverse biological pathways.

Sorghum is a diploid species (2n = 20) with a genome size of approximately 730 megabases (Mb), which makes it amenable to whole-genome sequencing [30]. In this study, we investigate the mutagenic effects of FN irradiation by the whole-genome re-sequencing of 40 mutagenized M_1_ lines of the reference genome line ‘BTx623’ and compare genetic variants at eight FN doses to determine the optimal dose of FN irradiation for sorghum. This study aims to provide insights into the mechanism of mutations induced by FN irradiation in sorghum, thereby accelerating the effective application of radiation mutagens in sorghum genomic research and breeding.

## 2. Results

### 2.1. Effect of FN Irradiation on Seed Germination

The average seed germination rate of the control group was 89%, and the average seed germination rate of the groups treated with 5 Gy, 11 Gy, 14 Gy, 19 Gy, 26 Gy, 33 Gy, 41 Gy and 46 Gy was 94%, 86%, 95%, 93%, 91%, 82%, 81% and 72%, respectively (Figure S1). Except for the group receiving 46 Gy irradiation, the differences in average seed germination rate between the control and the other groups were not significant.

### 2.2. Identification of Genome-Wide Mutations in FN Lines

We randomly selected five M_1_ seedlings from each treatment group for whole-genome resequencing using the Illumina high-throughput sequencing platform. On average, 108 million raw reads were obtained for each sample, with an average sequencing depth of 16× (the ratio of the total number of bases obtained by sequencing to the genome size) (Table 1). After a stringent quality filtering process, more than 15 Gb of high-quality clean data (Q30 > 91.75%) was retained for each sample. The filtered high-quality reads were aligned to the ‘BTx623’ reference genome. On average, more than 98% of the clean reads were successfully mapped to the reference genome. Approximately 98% of the reference sequences were mapped at least five times, and 91% of them were mapped at least ten times (Table 1).

We used an established variant-calling pipeline containing multiple complementary programs to call variants in each mutant after filtering out variants present in the unmutagenized sorghum. A total of 1491 FN-induced small mutations were identified in the 40 mutants, including 1342 SBSs and 149 InDels (Table S1). Of the 1342 SBSs, 25 were shared by the eight mutant lines. In total, 1406 FN-induced SVs were identified in the 40 mutants, including 504 large deletions, 384 duplications and 518 inversions. On average, 43.7 single-base substitutions (SBSs), 3.7 InDels and 35.15 SVs were detected in each sorghum individual.

### 2.3. Characteristics of the SBSs Induced by FN Irradiation

**Figure 1:**
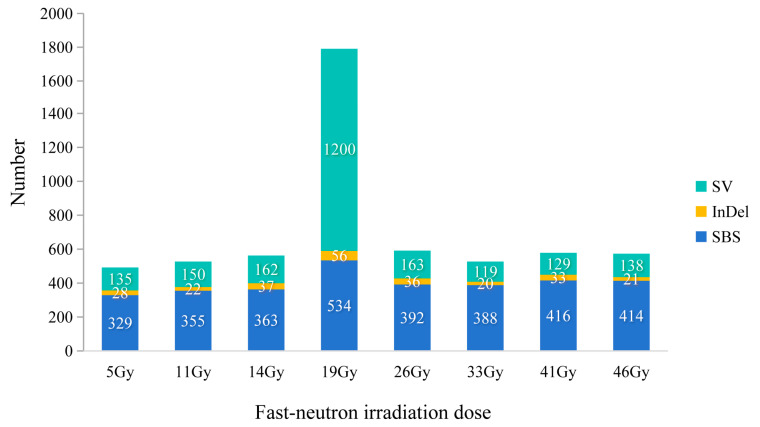
The number of each type of mutation in the eight treatment groups. SV, structural variation; InDel, insertion and deletion; SBS, single-base substitution.

**Figure 2:**
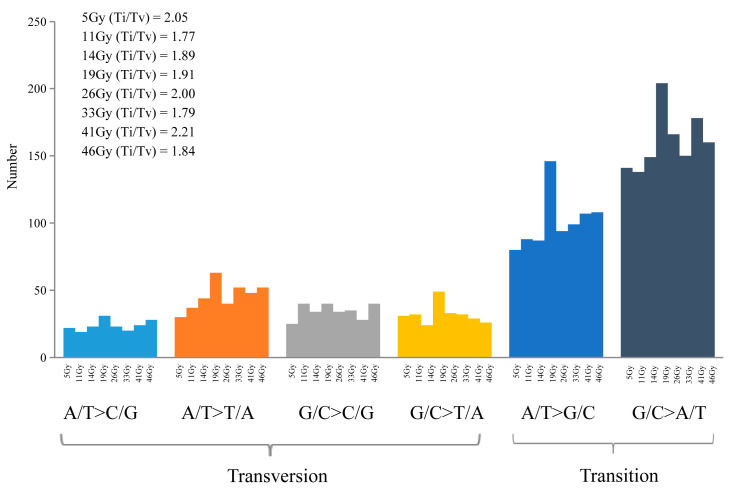
FN irradiation-induced transitions and transversions in the eight treatment groups. Ti, transitions; Tv, transversions.

To determine whether different doses of FN irradiation induced distinct mutations, we compared the genomic variants present in the eight mutant lines. SBSs accounted for approximately 90.0% of the total number of small mutations. The number of SBSs ranged from 329 to 534, with the 19 Gy-treated lines having the highest number of mutants (Figure 1). SBSs can be transitions (purine to purine; pyrimidine to pyrimidine) or transversions (purine to pyrimidine; pyrimidine to purine). FN irradiation induced both types of substitution, including A/T to G/G, G/C to A/T, A/T to C/G, A/T to T/A, G/C to T/A and G/C to C/G. The G/C to A/T transition was the most common substitution in all mutant lines (Figure 2). The ratio of Ti/Tv was the lowest in the 11 Gy-treated lines (~1.77) and the highest in the 41 Gy-treated lines (~2.21). A significant higher number of heterozygous mutations (average of 396.5 SBSs) than of homozygous ones (average of 2 SBSs) (*p* < 0.01) were detected in all mutant groups.

### 2.4. Characteristics of InDels and SVs Induced by FN Irradiation

**Figure 3:**
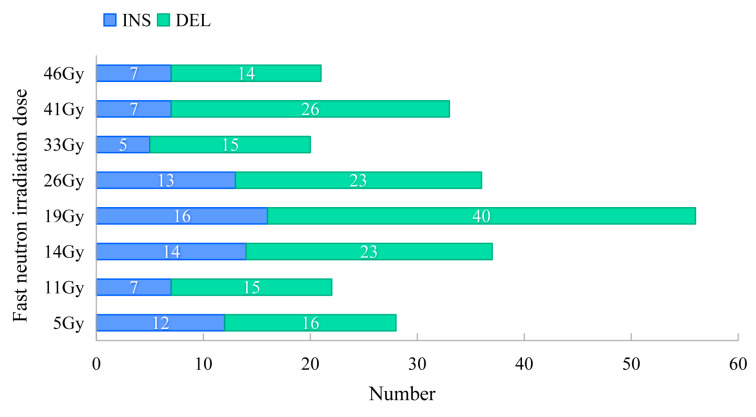
FN irradiation-induced InDels in the eight treatment groups. InDels, insertions and deletions; INS, insertion; DEL, deletion.

A total of 253 small InDels were detected on the genome-wide scale, of which 172 (68%) were deletion mutations, and 81 (32%) were insertions. The number of small deletions and insertions in the eight groups ranged from 15 to 40 and from 5 to 16, respectively. The highest proportion of InDels was detected in the 19 Gy-treated lines (Figure 3). The size of the FN-induced InDels ranged from 1 to 125 bp. Among the 172 small deletions, 91 (53%) single-base deletions and 81 (47%) multiple-base deletions (≥2 bp) were detected. Meanwhile, 37 (46%) of the 81 small insertions were single-base mutations, of which 44 (54%) had a size ≥2 bp. Single-base InDels were the most predominant type of InDels in the FN lines exposed to 5 Gy or to doses greater than 33 Gy, whereas multiple-base InDels (≥2 bp) were the most predominant type in 11~26 Gy-treated lines (Table S2). FN irradiation induced a significant higher number of heterozygous mutations (average of 26.75 InDels) than of homozygous ones (average of 4.88 InDels) (*p* < 0.01; two-side Student’s *t*-test) in all mutant groups. The number of FN-induced large-fragment deletions (>150 bp), duplications and inversions in the eight mutant lines ranged from 81 to 373, from 36 to 332 and from 1 to 494, respectively (Table S3). The highest number of SVs was found in the 19 Gy-treated lines, which was significantly higher than that in the other mutant groups (*p* < 0.01).

### 2.5. Distribution of Mutations Induced by FN Irradiation

**Figure 4:**
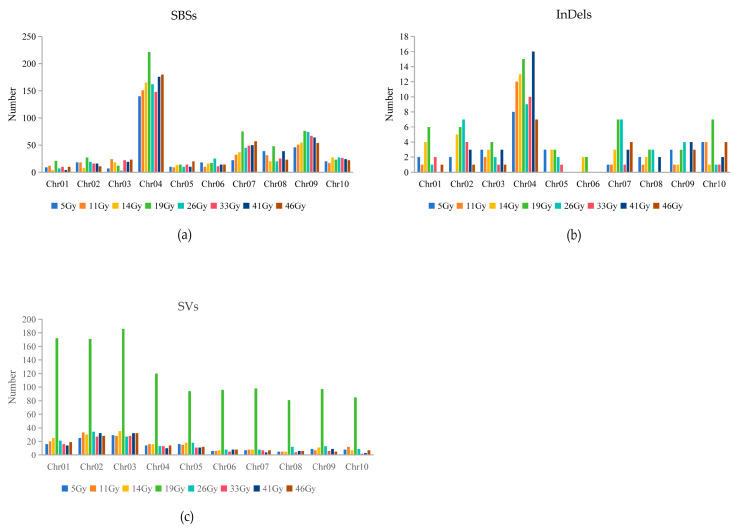
The number of SBSs, InDels and SVs in each chromosome induced by FN irradiation in the eight treatment groups. (**a**) FN irradiation-induced SBS mutations in each chromosome in the eight treatment groups. (**b**) FN irradiation-induced InDel mutations in each chromosome in the eight treatment groups. (**c**) FN irradiation-induced SV mutations in each chromosome in the eight treatment groups. SBSs, single-base substitutions; InDels, insertions and deletions; SVs, structural variations.

**Figure 5:**
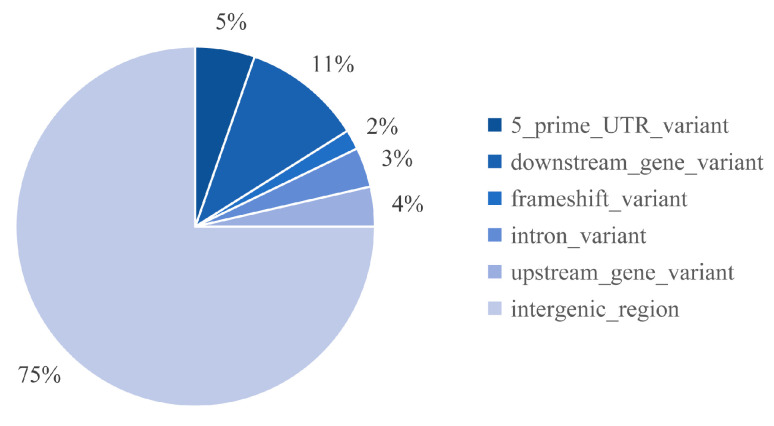
The distribution of InDel mutations across the genome in the 19 Gy-treated lines. InDel, insertion and deletion.

The distribution of SBSs and InDels in the sorghum chromosomes is shown in Figure 4. SBSs in each mutant affected all chromosomal segments. However, there were substantially more SBSs and InDels in some chromosomes than in others. For example, chromosome 4 had 42% and 38% of all SBSs and InDels (Figure 4a,b). Large-fragment deletions and duplications occurred more frequently in chromosome 2 and chromosome 3 (Figure 4c). The variants were divided into distinct categories according to their genomic locations. We found that the frequencies of SBSs in the genic regions were less than 3% in all mutant groups, significantly lower than in the intergenic regions (approximately 98%–100%) (Figure S2a). The frequencies of InDels in the genic regions were also lower than in the intergenic regions, ranging from 5% to 25%, with the highest frequency in the 19 Gy-treated lines (Figure S2b). Forty-two of the observed InDels in the 19 Gy-treated lines were located in intergenic regions, eight in downstream and upstream regions of genes, and six within untranslated regions (UTRs), with one change inducing a frameshift (Figure 5).

### 2.6. Genes Affected by FN Irradiation

Mutations located in the 5’UTR–3’UTR region may have an impact on gene function. A total of 19 genes were affected by 28 small mutations, with an average of 3.63 genes in each mutant line (Figure S3). SBSs and small deletions mutated 46% of the affected genes. Small insertions only affected two genes. Of the 19 genes, 6 were mutated in different mutant lines. Three of the nineteen genes produced more than two mutation types, especially in Sobic.004G197700, which produced eight types of mutation (Table 2). To further investigate the functions and pathways of the affected genes, all 19 genes were subjected to KEGG pathway and GO analyses. KEGG pathway analysis revealed that the affected genes were involved in 10 pathways, including the hippo signaling pathway, NADPH-ferrihemoprotein reductase pathway, glycerolipid metabolism, nucleocytoplasmic transport, pathways involving protein phosphatases and associated proteins, DNA repair and recombination, transfer RNA biogenesis, photosynthesis, ubiquitin-mediated proteolysis, exosome. GO annotation analysis showed that the 10 affected genes were enriched in 6 biological process terms, 3 cellular component terms, and 16 molecular function terms (Table S4).

## 3. Discussion

The radiation dose is an important factor affecting the effectiveness of radiation breeding. In this study, sorghum ‘BTx623’ seeds were treated with different doses of FN irradiation, and it was expected that the degree of damage to the M_1_ plants would increase with an increasing irradiation dose, and seed germination would tend to decrease. However, the germination survey showed that treatment of ‘BTx623’ seeds with 5–41 Gy of FN irradiation did not result in a decrease in the germination rate with an increasing treatment dose, but treatment with 46 Gy of FN led to a significant decrease in seed germination rate. This indicates that the damage induced by FN doses under 41 Gy did not affect the normal germination of sorghum seeds.

We sequenced a selection of 40 M_1_ mutants with an average of 16× coverage of the whole genome and identified a total of 2897 FN-induced mutations. The number of mutations per sequenced FN-mutagenized line ranged from 98 (5 Gy) to 358 (19 Gy), with an average of 141. Based on the 108 homozygous mutations detected in the M_3_ plants, Belfield et al. [13] estimated an original number of 288 heterozygous FN-induced mutations in the M_1_ plants of *A. thaliana*. The average number of mutations per individual was higher in sorghum than in *A. thaliana*, which may be related to sorghum larger genome. The dose that leads to 50% lethality (LD50) is usually considered an appropriate dose. LD50 might lead to a high number of (mostly deleterious) mutations, but some mutations are likely to be lost or overlooked in generations following the mutagenesis due to either plant mortality or poor agronomic performance. Among the different dose treatments, our study found that 19 Gy FN irradiation induced the highest number of mutations and did not lead to a reduction in the germination rate. Maluszynski et al. [31] suggested that the final doses for a mutagenic treatment should be rather low if the aim is to add new traits to an already high-quality genetic background. The present study confirms that a moderate radiation dose and a survival rate of 90% can induce a large number of mutations and shows that 19 Gy FN irradiation appears to be the most suitable dose for sorghum mutation breeding.

Traditionally, FN mutagenesis was thought to induce large (300 bp–8 kb) deletion mutations. For example, large deletions and duplications were detected in a soybean FN-irradiated mutant population [32]. However, in *A. thaliana* and rice, FN-induced SBSs were found to be the most abundant mutation type in genome-sequenced M_3_ lines, and small deletions (<10 bp) were more prevalent than larger deletions [13,14]. The analysis of sorghum M_1_ lines showed that FN irradiation induced a similar number of small mutations and structural variations. In addition, unlike in *A. thaliana* and rice, the proportions of single-base and ≥2-base insertions and deletions were similar among the 172 InDels in sorghum, with the overall abundance of small deletions being twice as high as that of insertions. This discrepancy may be due to the fact that some large deletion mutations that are not transmitted to the progeny [33] or may result in lethality when homozygous were under-represented in the M_3_ lines.

Although SBS was the second most common type of mutation induced by FN irradiation in sorghum, it was much less frequent than in mutants induced by EMS. Simons et al. [28] sequenced an EMS-induced sorghum mutant population and identified 3,274,606 pf SNPs. Xin et al. [34] detected more than 1.8 million canonical EMS-induced mutations in 252 sorghum mutants. Jiao et al. [35] found that as the sequencing depth doubled, the number of mutations detected in each mutant line also doubled. Therefore, we could improve mutation detection by increasing the sequencing depth in subsequent studies. EMS mainly induces G/C to A/T DNA substitutions, which usually account for more than 95% of all SBSs it causes [36,37]. In wheat, the percentage of γ ray- and C-ion beam-induced G/C to A/T transitions was found to be approximately half of that detected in EMS mutant lines [35]. Similarly, we detected that the percentage of FN-induced G/C to A/T transitions was 40%, suggesting that physical mutagens (e.g., fast neutrons, γ rays and ion beams) induce more diverse mutations than EMS [38].

The distinct characteristics of physical/chemical mutagenesis lead to different distribution patterns of mutations. For example, chemically induced single-nucleotide variants were found to be almost uniformly distributed across the *Caenorhabditis elegans* genome [39]. In contrast, Liu et al. [40] detected an uneven distribution of radiation-induced SBSs across the cotton genome. In this study, FN-induced mutations were also unevenly distributed across the sorghum genome, and the number of variants was significantly higher in some chromosomes than in others. For example, SBSs and InDels in chromosome 4 accounted for 42% and 38% of all SBSs and InDels, whereas large-fragment deletions and duplications occurred more frequently in chromosomes 2 and 3. An uneven distribution of mutations may be caused by two factors. On the one hand, it may be related to genome features. Monroe et al. [41] revealed that the natural mutation bias is associated with GC content, methylated cytosines and gene structure. Weng et al. [42] found that the uneven distribution of spontaneous mutations in *A. thaliana* genome favored G: C to A: T transitions. Similarly, we showed that the G/C to A/T transition was the most common substitution in all mutant lines of sorghum.

In addition, we found that the mutations were mostly distributed in intergenic regions, with fewer mutations in gene regions. This observation suggests that mutations in sorghum occur preferentially in non-coding regions, and local differences in chromatin architecture might differentially affect DNA repair mechanisms and thus the local mutation rate. On the other hand, the observed uneven distribution of mutations could be related to the properties of fast-neutron radiation. High-LET particles like fast neutrons produce a strong local ionization on their penetrating path. It was found that the action of high-LET rays on cells produces a certain density of uneven energy deposition in DNA molecules and induces several local DNA damage clusters. Such localized multiple damage sites may affect the distribution of mutants [43,44].

## 4. Materials and Methods

### 4.1. Plant Material and Generation of the Mutant Library

The first sequenced inbred line, ‘BTx623’, was used to establish thane FN-induced mutant library. Dry seeds were irradiated with FNs at doses of 5 Gy, 11 Gy, 14 Gy, 19 Gy, 26 Gy, 33 Gy, 41 Gy and 46 Gy at the China institute of atomic energy. The ray energy was set to 14 MeV. The treated seeds were planted, and the resulting M_1_ plants were grown in the Liuhe base of the Jiangsu Academy of Agricultural Sciences in 2023. Seeds from individual M_1_ plants were collected and stored.

### 4.2. Germination Test

For each treatment and control group, three replicates of 50 sorghum seeds were placed in plastic germination boxes (13 cm × 19 cm × 9 cm) containing four layers of filter paper moistened with sterile water and grown in a growth chamber under a 12/12 h day/night light cycle and at 25 °C. The germination rate was calculated as (number of seeds showing radicle emergence (2 mm in length) after 3 days of incubation/total number of seeds) × 100%.

### 4.3. Whole-Genome Re-Sequencing

The young leaves of five M_1_ plants were randomly collected from each treatment group and the control group, and five plants from the control group were mixed into one sample. DNA extraction was conducted using the DNeasy Plant Maxi Kit (Qiagen Inc., Valencia, CA, USA) according to the manufacturer’s protocol. The DNA libraries were prepared with the TruSeq DNA Sample Preparation Kit (Illumina Inc.). Paired-end (2·150 bp) sequencing was performed on Illumina NovaSeq 6000 (Illumina, San Diego, CA, USA) to determine genomic sequences with about 16-fold depth for each line (LC-Bio, Hangzhou, Zhejiang Province, China).

### 4.4. Genomic Variant Detection and Annotation

After removing low-quality reads with adaptor sequences, the clean data were used for further bioinformatics analysis. The clean data were mapped to the reference genome Sorghum bicolor v3.1.1 (https://phytozome-next.jgi.doe.gov/info/Sbicolor_v3_1_1) (accessed on 6 July 2023) using BWA with the default parameters [45]. SAMtools software (v.1.3) was used to convert, index and sort the mapping results to BAM files. If multiple read pairs had identical external coordinates, only the pair with the highest mapping quality was retained. Duplicate reads were removed using Picard MarkDuplicates (v1.94). GATK HaplotypeCaller v4.0.4 was used to call single-base substitutions (SBSs) and InDels. SNPs were filtered using VariantFiltration with QD < 2.0, FS > 60.0, MQ < 40.0, SOR > 3.0, MQRankSum < −12.5, ReadPosRankSum < −8.0, QUAL < 30.0, and InDels with QD < 2.0, FS > 200.0, QUAL < 30.0, ReadPosRankSum < −8.0. The variations were annotated by SnpEff software (v4.3). Lumpy [46] was used to call SVs. Samples from each treatment group were analyzed in groups of five mutant lines and the control line. Only those mutation sites that differed from mutation sites in both the reference genome and the control line were considered reliable mutation sites. GO enrichment and KEGG pathway analyses were performed to identify the functions of the affected genes [47,48]. *p* < 0.05 was set as the cutoff criterion for significant enrichment.

### 4.5. Statistical Analysis

The data were subjected to analysis of variance (ANOVA) using SPSS 18.0 statistical software to compare germination rates and number of mutations in the different treatment groups. The significance level was set at *p* < 0.05.

## 5. Conclusions

The present study investigated the genetic mutations induced by FN irradiation in sorghum. The results revealed that FN mutagenesis is a powerful tool for sorghum breeding as it can produce a moderate amount and multiple types of mutations compared to other mutagens such as EMS. The appropriate dose of FN identified in this study can be beneficial for plant breeders for further dose optimization and sorghum large-scale mutation breeding programs, as well as for breeding programs for other crops. In the future, we will conduct high-throughput phenotypic and genomic analyses of FN-irradiated M_2_ mutant populations to deepen our understanding of the mechanisms of radiation mutagenesis and its application in gene function analysis.

## Figures and Tables

**Table 1:** Statistics of the whole-genome re-sequencing data of the 41 sorghum samples.

Mutant ID	Number of Raw Reads	Number of Clean Reads	Number of Clean Bases (Gb)	Mapping Rate (%)	Depth of Coverage ≥ 20× (%)	Depth of Coverage ≥ 10× (%)	Depth of Coverage ≥ 5× (%)	Q20%	Q30%	GC%
Bs5_1	105691374	103875740	15.51	98.19	43.79	92.33	98.91	97.02	92.32	43.52
Bs5_2	108522048	106884932	15.96	98.71	47.20	93.62	99.08	97.17	92.65	43.34
Bs5_3	105939082	104319734	15.57	97.51	42.28	92.51	98.98	97.19	92.66	43.54
Bs5_4	98457400	97053746	14.49	97.22	34.18	89.73	98.69	97.21	92.76	43.69
Bs5_5	107326540	105233474	15.72	97.97	44.45	93.04	99.03	96.89	92.12	43.57
Bs11_1	107332754	105249272	15.71	97.79	46.33	89.45	97.36	96.91	92.23	44.78
Bs11_2	105666644	103826436	15.51	98.03	43.64	92.58	98.99	96.98	92.24	43.52
Bs11_3	106434116	104599640	15.62	98.47	44.95	92.93	98.98	96.99	92.26	43.46
Bs11_4	107693462	105623622	15.77	98.18	44.03	92.91	99.02	96.89	92.13	43.42
Bs11_5	105326402	103602506	15.46	98.51	43.76	92.80	98.99	97.04	92.33	43.44
Bs14_1	108693522	106686736	15.93	98.38	45.76	93.19	99.04	96.95	92.29	43.38
Bs14_2	109430936	107883362	16.1	98.64	48.44	93.89	99.12	97.21	92.87	43.37
Bs14_3	107017144	105162890	15.7	98.37	45.48	92.85	99.01	97	92.3	43.41
Bs14_4	107153690	105324132	15.73	98.03	44.67	92.73	98.99	97.01	92.32	43.46
Bs14_5	104585328	103151406	15.39	98.65	42.51	92.33	98.96	97.25	92.98	43.29
Bs19_1	108469448	106800680	15.94	97.45	45.40	93.48	99.11	97.17	92.89	43.59
Bs19_2	108122626	106404360	15.89	98.34	45.61	93.56	99.12	97.1	92.49	43.39
Bs19_3	108469448	106800680	15.94	97.45	45.40	93.48	99.11	97.17	92.89	43.59
Bs19_4	107796868	105588026	15.77	98.4	45.48	93.44	99.10	96.85	92.04	43.43
Bs19_5	108507344	106160820	15.85	98.52	46.64	93.43	99.08	96.69	91.78	43.41
Bs26_1	109657890	108202156	16.14	98.61	47.79	94.23	99.13	97.52	93.47	43.22
Bs26_2	108054944	105624632	15.78	98.28	46.02	93.43	99.08	96.7	91.75	43.51
Bs26_3	109460390	107190882	16.01	98.29	47.89	94.05	99.15	96.76	91.92	43.45
Bs26_4	108326044	106128352	15.84	97.8	45.16	93.36	99.09	96.81	91.93	43.49
Bs26_5	108149020	106462900	15.89	98.85	48.22	94.08	99.17	97.16	92.79	43.35
Bs33_1	108844444	107644812	16.05	97.03	45.41	89.30	97.11	97.6	93.74	44.87
Bs33_2	109326180	107551264	16.04	98.18	46.63	90.42	97.59	97.25	92.95	44.43
Bs33_3	109850900	108089326	16.12	98.48	49.80	91.11	97.66	97.25	92.92	44.57
Bs33_4	108885036	107371884	16.02	98.65	48.37	91.61	97.97	97.41	93.24	44.24
Bs33_5	108389176	106722290	15.93	98.62	46.88	94.00	99.15	97.36	93.07	43.22
Bs41_1	108968604	107382338	16	98.18	48.31	90.02	97.24	97.34	93.1	44.75
Bs41_2	108959848	107167034	15.98	94.38	43.29	87.89	96.80	97.18	92.77	45.56
Bs41_3	107782964	106085224	15.83	98.29	44.79	88.96	97.21	97.24	92.82	44.56
Bs41_4	109794748	107912634	16.1	98.38	47.03	90.68	97.71	97.13	92.67	44.38
Bs41_5	107973556	105970066	15.81	97.55	45.69	89.84	97.45	97.03	92.47	44.64
Bs46_1	107332754	105249272	15.71	97.79	46.33	89.45	97.36	96.91	92.23	44.78
Bs46_2	108282810	106441380	15.88	98.71	46.43	88.89	97.03	97.09	92.54	44.64
Bs46_3	107892514	106163432	15.83	98.19	46.04	88.70	96.96	97.17	92.69	44.79
Bs46_4	105055526	103510568	15.44	98	44.71	88.34	96.98	97.27	93.09	44.8
Bs46_5	108503148	106871610	15.94	98.18	45.50	88.57	96.84	97.29	92.95	44.67
BTx623	108293796	106194916	15.86	99.01	48.73	93.69	99.11	97.12	92.56	43.42
Mean	107668792	105857785	15.80	98.10	45.59	91.83	98.40	97.10	92.59	43.90

**Table 2:** Mutation information regarding the genes affected by small mutations.

Line	ID	Chr	Pos	Ref	Alt	GeneID
5 Gy	Chr01_62221922	Chr01	62221922	G	GGC	Sobic.001G299900.v5.1
	Chr04_57358919	Chr04	57358919	TCGA	T	Sobic.004G197700.v5.1
	Chr04_57358941	Chr04	57358941	C	T	Sobic.004G197700.v5.1
	Chr04_57358981	Chr04	57358981	A	T	Sobic.004G197700.v5.1
	Chr09_28770284	Chr09	28770284	T	G	Sobic.009G095560.v5.1
11 Gy	Chr03_6409060	Chr03	6409060	CA	CAA,C	Sobic.003G074700.v5.1
14 Gy	Chr01_62221922	Chr01	62221922	G	GGC	Sobic.001G299900.v5.1
	Chr01_10357147	Chr01	10357147	T	A	Sobic.001G130400.v5.1
	Chr02_48202647	Chr02	48202647	TCA	T	Sobic.002G155500.v5.1
19 Gy	Chr02_48202647	Chr02	48202647	TCA	T	Sobic.002G155500.v5.1
	Chr03_70852413	Chr03	70852413	CAG	C	Sobic.003G317500.v5.1
	Chr03_79824423	Chr03	79824423	AG	A	Sobic.003G431800.v5.1
	Chr04_28733502	Chr04	28733502	G	A	Sobic.004G135383.v5.1
	Chr04_28733574	Chr04	28733574	A	T	Sobic.004G135383.v5.1
	Chr05_6918726	Chr05	6918726	C	T,G	Sobic.005G062200.v5.1
	Chr05_10737537	Chr05	10737537	TC	T	Sobic.005G080062.v5.1
	Chr06_58090348	Chr06	58090348	ACTCT	A,ACT	Sobic.006G219600.v5.1
	Chr07_97864	Chr07	97864	CGAGA	CGA,C	Sobic.007G000900.v5.1
26 Gy	Chr01_30720439	Chr01	30720439	T	C	Sobic.001G257400.v5.1
	Chr01_62221922	Chr01	62221922	G	GGC	Sobic.001G299900.v5.1
	Chr02_48202647	Chr02	48202647	TCA	T	Sobic.002G155500.v5.1
	Chr02_68565702	Chr02	68565702	TC	T	Sobic.002G295100.v5.1
	Chr08_62900559	Chr08	62900559	T	A	Sobic.008G164526.v5.1
	Chr09_24008650	Chr09	24008650	T	C	Sobic.009G092900.v5.1
33 Gy	Chr01_62221922	Chr01	62221922	G	GGC	Sobic.001G299900.v5.1
41 Gy	Chr04_57358919	Chr04	57358919	TCGA	T	Sobic.004G197700.v5.1
	Chr04_57358988	Chr04	57358988	CGGG	C	Sobic.004G197700.v5.1
	Chr04_57358993	Chr04	57358993	ACGG	A	Sobic.004G197700.v5.1
	Chr04_57359003	Chr04	57359003	CGAT	C	Sobic.004G197700.v5.1
	Chr04_57358941	Chr04	57358941	C	T	Sobic.004G197700.v5.1
	Chr04_57358981	Chr04	57358981	A	T	Sobic.004G197700.v5.1
	Chr05_6918726	Chr05	6918726	C	T,G	Sobic.005G062200.v5.1
	Chr08_62900559	Chr08	62900559	T	A	Sobic.008G164526.v5.1
	Chr08_62900574	Chr08	62900574	A	G	Sobic.008G164526.v5.1
46 Gy	Chr01_62221922	Chr01	62221922	G	GGC	Sobic.001G299900.v5.1
	Chr01_73368414	Chr01	73368414	A	G	Sobic.001G408300.v5.1
	Chr04_57358833	Chr04	57358833	G	A	Sobic.004G197700.v5.1
	Chr04_57358850	Chr04	57358850	TG	T	Sobic.004G197700.v5.1
	Chr10_60900949	Chr10	60900949	CAG	C	Sobic.010G254300.v5.1

## Data Availability

Data are contained within the article and Supplementary Materials.

## Supplementary Materials

The following supporting information can be downloaded at: https://www.mdpi.com/article/10.3390/genes15080976/s1, Figure S1: The germination rate of sorghum seeds at different FN irradiation doses. NS, not significant; *, *p* < 0.05; Figure S2: (a) The frequencies of SBSs in genic and intergenic regions; (b) the frequencies of InDels in genic and intergenic regions.; Figure S3: The number of genes affected by FN irradiation in each treatment group; Table S1: Information on 1491 FN-induced small mutations in the 40 mutants; Table S2: Number of different types of InDels induced by FN irradiation.; Table S3: Number of structural variations induced by FN irradiation; Table S4: KEGG pathway and GO analyses of genes affected by FN irradiation.
