# Supplementary figures and images for: Comparison of Mutations Induced by Different Doses of Fast-Neutron Irradiation in the M1 Generation of Sorghum (Sorghum bicolor)
Source: Genes (Basel). 2024 Jul 24;15(8):976. doi: 10.3390/genes15080976 (PMC11354182; doi:10.3390/genes15080976)

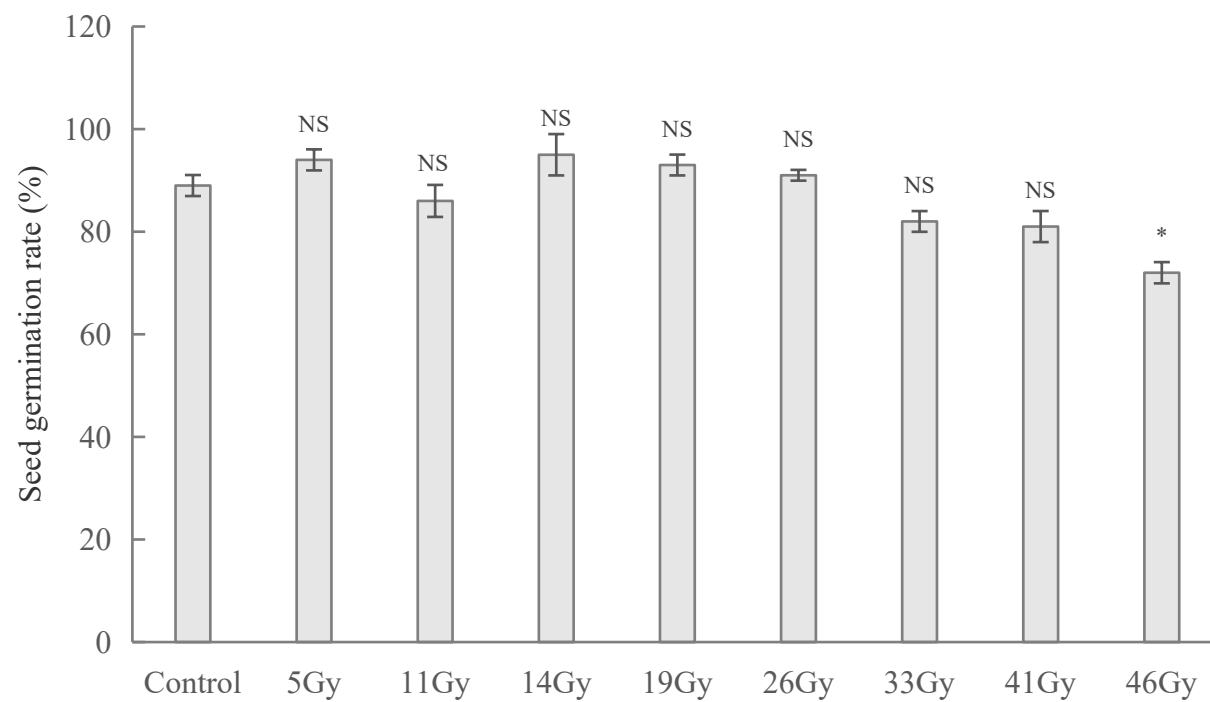

Supplement: Supplementary file 1 [file genes-15-00976-s001.zip › Figure S1.pdf]

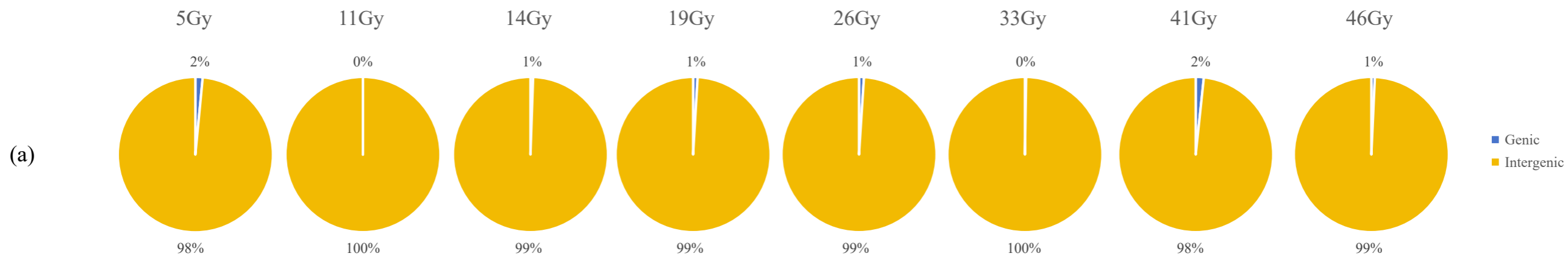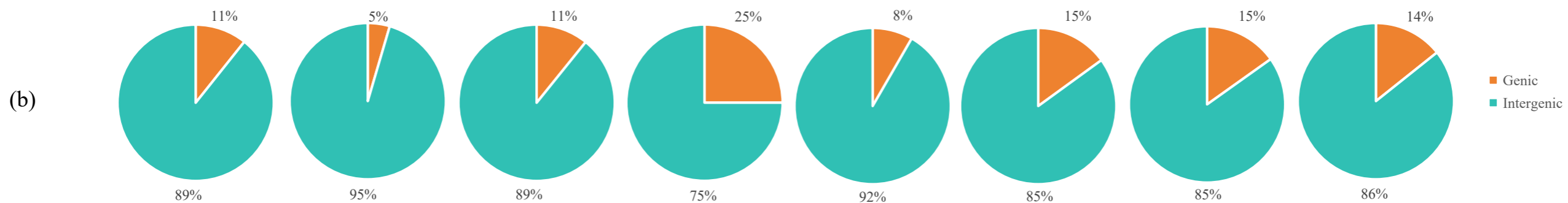

Supplement: Supplementary file 1 [file genes-15-00976-s001.zip › Figure S2.pdf]

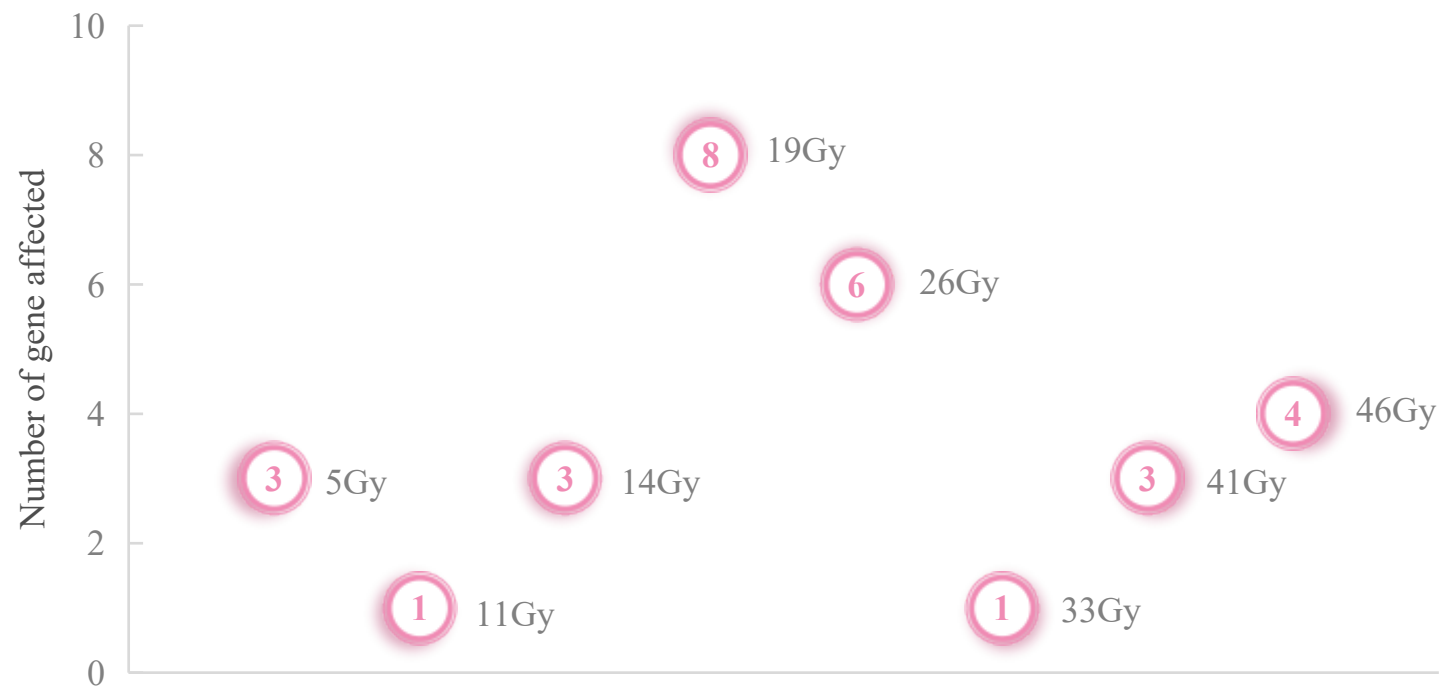

Supplement: Supplementary file 1 [file genes-15-00976-s001.zip › Figure S3.pdf]
